# Transcriptome Analysis of Human Vascular Smooth Muscle Cells Cultured on a Polyglycolic Acid Mesh Scaffold

**DOI:** 10.1155/2023/9956190

**Published:** 2023-06-22

**Authors:** Jiang Liu, Zibei Feng, Peng Liu, Lijun Fang, Xichun Wang, Haiyan Lao, Yueheng Wu, Zhanyi Lin

**Affiliations:** ^1^Department of Pharmacy, Guangdong Provincial People's Hospital (Guangdong Academy of Medical Sciences), Southern Medical University, Guangzhou, Guangdong 510080, China; ^2^Ji Hua Institute of Biomedical Engineering Technology, Ji Hua Laboratory, Foshan, Guangdong 528200, China; ^3^School of Medicine, South China University of Technology, Guangzhou, Guangdong 510006, China; ^4^Guangdong Cardiovascular Institute, Guangdong Provincial People's Hospital (Guangdong Academy of Medical Sciences), Southern Medical University, Guangzhou, Guangdong 510080, China; ^5^Guangdong Provincial Key Laboratory of South China Structural Heart Disease, Guangdong Provincial People's Hospital (Guangdong Academy of Medical Sciences), Southern Medical University, Guangzhou, Guangdong 510080, China; ^6^Guangdong Provincial Geriatrics Institute, Guangdong Provincial People's Hospital (Guangdong Academy of Medical Sciences), Southern Medical University, Guangzhou, Guangdong 510080, China

## Abstract

To construct tissue-engineered blood vessels (TEBVs) *in vitro*, it is necessary to transfer seed cells to three-dimensional (3D) scaffolds for culture. However, what happens to the behavior of the cells after they are transferred to the scaffold is unclear. Therefore, in this study, a transcriptome analysis was used to characterize the differentially expressed genes (DEGs) of vascular smooth muscle cells (VSMCs) before and after transfer to 3D polyglycolic acid (PGA) scaffolds and to understand the changes in functional gene expression in the early stage of 3D culture. Transcriptome sequencing results showed that DEGs in the seed cells were mainly enriched in cell proliferation and cell-cell adhesion. The DEGs of cells grown in a 3D PGA scaffold (PGA-VSMCs) were mainly enriched in signal transduction. Furthermore, we found that ERK1/2 was significantly activated in PGA-VSMCs and inhibiting the phosphorylation level of ERK 1/2 in PGA-VSMCs significantly increased the expression of elastin. In conclusion, the PGA scaffold material altered gene expression in VSMCs and affected the elastin production. This study advances our understanding of biomaterial-cell interactions and provides valuable insights for improving the cultivation of TEBVs.

## 1. Introduction

Tissue-engineered blood vessels (TEBVs) are regarded as the most promising treatment for severe coronary and peripheral arterial disease that is medication refractory. Several teams have made outstanding contributions to the field of TEBVs [1, 2]. The team of Kirkton developed an investigational bioengineered blood vessel generated by seeding human vascular smooth muscle cells (VSMCs) into a biodegradable polyglycolic acid (PGA) mesh scaffold within a bioreactor system [3]. Through the automated system for the production of engineered vessels for mass production [4], it is currently being studied as a hemodialysis catheter in patients with an end-stage renal disease. Our team successfully constructed TEBVs using PGA scaffolds [5, 6]. However, the secretion of extracellular matrix (ECM) is still not satisfactory to us. Dahl's team found a similar situation, with TEBVs being rich in collagen but lacking in elastin [7].

The research protocol for the *in vitro* construction of TEBVs is to seed the cells into the scaffold material. The long-term cultures are then performed in a bioreactor system with cyclic stretching and medium circulation. Finally, a decellularization process is performed to eliminate immunogenicity [8–10]. In the preparatory work for culture, seeded cells need to be isolated and passaged to reach the desired number and then transferred to scaffolds. To clarify which genes are altered after seeding cells on scaffolds, the transcriptome sequencing technology offers a solution. Klapperich and Bertozzi infused human fibroblasts into 3D collagen-glycosaminoglycan scaffolds and used transcriptomics to compare the scaffold cell gene expression with the seed cells. It was found that genes involved in cell transduction, ECM protein restructuring, inflammation, angiogenesis, and oxygenation were activated [11]. Jager et al. found that 3D VSMC aggregates can reduce the expression of genes related to cell cycle control and DNA replication and cease the proliferation of VSMC [12]. Lin et al. found that VSMCs made in 3D alginate hydrogel tubes had higher expression of genes related to vasculature development and angiogenesis [13]. By detecting the expression of all genes, researchers can comprehensively assess all signaling pathways related to cell behavior, thereby gaining information on the molecular mechanisms of biomaterials that stimulate various cellular behaviors.

The ultimate goal of TEBVs is to create replacements for natural arteries. Only in the early stage of culture can seed cells secrete sufficient ECM to produce vascular grafts with the required mechanical properties [14]. In native blood vessels, elastin provides elasticity to the vascular wall, allowing blood vessels to reversibly expand and contract [15]. Furthermore, elastin fibers are known to promote maintenance of the contractile phenotype of VSMCs, which is critical to prevent overproliferation in TEBVs [16, 17]. The lack of sufficient elastin synthesis and assembly into fibrils remains an unsolved problem in TEBVs [18]. Other studies suggested that in an *in vitro* engineered setting, the greatest obstacle to elastic tissue generation may be reduced tropoelastin secretion, but essentially unchanged the levels of type I collagen synthesis [19]. Therefore, another goal of ours is to explore how the PGA scaffold material affects the secretion of elastin by VSMCs.

In this study, we analyzed the expression of vascular smooth muscle cells (seed cells) before and after the cells were grown on 3D PGA scaffold (PGA-VSMCs) for 4 days. Then, we performed a hierarchical cluster analysis on the differentially expressed genes and analyzed the function and pathway of each cluster. The authenticity of the transcriptome sequencing data was verified by qPCR and Western blotting. Finally, we attempted to explain the mechanism of the PGA scaffold that affected elastin secretion. Our research helps in gaining a better understanding of how VSMCs survive on PGA scaffolds and have a guiding significance for TEBV cultures.

## 2. Materials and Methods

### 2.1. Scaffold Preparation

The PGA mesh scaffold material (15 *μ*m in diameter, 1 mm thickness; Synthecon, Houston, TX, USA) was cut into a 1 × 1 cm rectangle. Then, the PGA was soaked in 1 M NaOH for 1 min and washed twice with sterile water for 2 min each time. The treated PGA scaffold was dipped in 75% alcohol for 2 h, and the residual alcohol was absorbed with a sterile cotton swab and then ventilated on an ultraclean bench for 2 h and dried for the subsequent analysis.

### 2.2. Gel Permeation Chromatography

Waters-Wyat gel permeation chromatography instrumentation was used to determine the molecular weight and distribution of the polymers. The gel permeation chromatography instrument was equipped with a 1515 high-performance liquid chromatography pump, a 2414 refractive index detector, two ShodexKF series columns, and hexafluoroisopropanol containing 3 mg/mL potassium trifluoroacetate as an elution agent. The flow rate was 0.8 mL/min, the temperature was 40°C, and the standard sample was polymethyl methacrylate.

### 2.3. Degradation Experiment

PGA samples (∼10 mg) were placed in tubes containing 5 mL of PBS buffer with the addition of 1% (v/v) penicillin/streptomycin in an incubator at 37°C and 5% CO_2_ for degradation experiments. On days 1, 4, 7, 10, 13, 16, 19, and 22, the liquid portion was removed from the test tube, and the test tube containing the undissolved polymer fibers was lyophilized. Each test was repeated at least three times. The weight of the remaining polymer (*W*_*t*_) was measured, and with the initial weight of the polymer (*W*_0_), the degradation was calculated using the following formula:

(1) $$\begin{array}{c} Mass\;remaining\left(\%\right)=\frac{w_{t}}{w_{0}}\times 100\%\text{.} \end{array}$$

### 2.4. Cell Culture

The protocols for the acquisition of human aortas from healthy donors were approved by the Guangdong General Hospital Research Ethics Committee (Project Code No. GDREC2018225H(R1)). All of the participants in the research provided their informed consent. VSMCs were obtained by standard explant techniques. The VSMC medium contained F-12K basal medium (Gibco, USA, Cat.No.c11330500), 20% fetal bovine serum (Gibco, USA, Cat.No 10099141C), and 100 U/mL penicillin and streptomycin (Gibco, USA, Cat.No. 15140122). The cells were grown at 37°C in 5% CO_2_, passed when fusion occurred, and collected for use within 3–6 generations.

The PGA scaffold was placed on a low-adhesion 24-well plate, and cell suspensions (1 × 10^6^ cells/mL) were transferred onto the PGA scaffold. The well plate was inverted for 4 h to allow the cells to adhere to the PGA scaffold. The scaffold was placed in a low-adhesion 6-well plate for subsequent culture.

### 2.5. Transcriptomic Sequencing and Data Analysis

The transcriptome sequencing analysis was performed on seed cells and PGA-VSMCs after 4 days of culture. TRIzol (Invitrogen, CA, USA Cat.No. 15596026) was used to isolate and purify the RNA from the entire sample, and the quantity and purity of the total RNA was evaluated. The product was purified, and the mRNA was fragmented using divalent cations at a high temperature, followed by PCR to create a library with the fragment sizes of 300 bp ± 50 bp. Finally, we performed paired-end sequencing using an Illumina Novaseq™ 6000 (LC BioTechnology Co., Ltd., Hangzhou, China) following normal procedures, using PE150 as the sequencing mode.

After using the Illumina paired-end RNA-seq method for transcriptome sequencing, FastQC software was used to perform quality control on the offline raw data, by removing adapters, repetitive sequences, and low-quality sequences. Default parameters were used. HISAT2 was used to compare the sequencing data to the genome (*Homo sapiens*, GRCh38), and FPKM was used to assemble and quantify the genes.

### 2.6. Identification of Differentially Expressed Genes and Bioinformatics Analysis

The R software tool DESeq2 was used to identify differentially expressed genes (DEGs). The genes with a *p* value <0.05 and |log_2_ FC| ≥ 1.2 were considered DEGs. To examine protein-protein interaction (PPI) information and cluster analysis, we removed outliers and employed the Search Tool for the Retrieval of Interacting Genes Database (STRING). We also utilized the Cytoscape tool cytoHubba to compute the rank of hub genes to investigate the link between DEGs. Gene Ontology (GO) enrichment and Kyoto Encyclopedia of Genes and Genomes (KEGG) analyses were performed using the Database for Annotation, Visualization, and Integrated Discovery (DAVID).

### 2.7. Immunofluorescence Staining

The samples were immersed in 4% paraformaldehyde and then penetrated with 0.2% Triton X-100 for 5 min and washed three times with PBS. A subset of the samples were treated with phalloidin (Proteintech, USA, 1 : 100, Cat.No.PF00003) for 20 min at room temperature before mounting on slides with DAPI (Thermo Fisher Scientific, USA, Cat.No.R37606). The other samples were blocked for 45 min at room temperature and diluted primary antibody was added (Elastin, Novus Biologicals, USA, 1 : 100, Cat.No.NB100-2076; *α*-SMA, Proteintech, USA, 1 : 100, Cat.No.67735-1-IG; calponin, Proteintech, USA, 1 : 100, Cat.No 13938-1-AP) and incubated at 4°C overnight. After washing, the corresponding secondary antibody was added. The samples were observed using a fluorescent confocal laser scanning microscope.

### 2.8. Scanning Electron Microscopy

The samples were fixed for 10 min in a 2.5% glutaraldehyde solution, rinsed for 5 min with distilled water, and dehydrated for 5 min in 70, 85, and 95% of absolute ethanol. The samples were then soaked for 10 min in hexamethyldisilazane and air-dried for 3 h. The dried samples were mounted, sputter-coated in gold (EMS150TES thin-film deposition machine), and examined with a field emission scanning electron microscope (SEM, Merlin Carl Zeiss Jena, Jena, Germany).

### 2.9. RNA Isolation and qRT-PCR Analysis

To extract total RNA from the samples, TRIzol (Invitrogen, USA, Cat.No.15596026) was employed. The acquired RNA was resuspended in RNase-free water. Then, an ultramicroscopic spectrophotometer was used to assess the purity and concentration of total RNA (Nanodrop 2000, Thermo Fisher Scientific, USA). Next, 1 *μ*g of RNA was reverse-transcribed into cDNA using a PrimeScript™ RT Reagent kit with gDNA Eraser (Takara, Tokyo, Japan, Cat.No.RR047A). The cDNA was mixed with TB Green® Premix Ex Taq™ (Takara, Tokyo, Japan, Cat.No.RR820A) and primers for measuring the expression of the target genes. *GAPDH* was used as a housekeeping gene. The primer sequences are listed in Table S1.

### 2.10. Western Blotting Analysis

The total protein was extracted from the sample using a total protein kit (BestBio, China, Cat.No.BB3101). SDS-PAGE (10%) was used to separate an equal quantity of protein from each sample, which was then transferred to a 0.22 *μ*m PVDF membrane (Millipore, USA, Cat.No.ISEQ10100). Subsequently, the membrane was blocked for 1 h with TBS containing 5% milk and 0.1% Tween 20 and then incubated overnight at 4°C in the primary antibodies elastin (Novus Biologicals, USA, 1 : 1000, Cat.No.NB100-2076), ERK1/2 (CST,USA, 1 : 1000, Cat.No.4695), phosphorylated ERK1/2 (CST, USA, 1 : 1000, Cat.No.4370), and GAPDH (Proteintech, USA, 1 : 10,000, Cat.No.60004-1-Ig). The membrane was incubated at 4°C for 2 h with goat antirabbit horseradish peroxidase (Abbkine, China, 1 : 10,000, Cat.No.A21020) or goat antimouse horseradish peroxidase (Abbkine, China, 1 : 10,000, Cat.No.A21010). Finally, we used an enhanced chemiluminescence reagent (BioSharp, China, Cat.No.69030060) to detect the protein bands and used an ImageQuant LAS 500 (Massachusetts, USA) for visualization. The intensity (gray value) of each protein band was calculated using ImageJ software, and GAPDH was utilized as a standardized reference.

### 2.11. Cell Viability Assay

Cell Counting Kit-8 (CCK-8) assays (Beyotime, China, Cat.No.C0038) were used to determine the viability of VSMCs according to the manufacturer's recommendations. In a 96-well plate, the PGA was cut into 0.25 × 0.25 cm squares. After treatment, VSMCs were seeded at a density of 8000 cells per well in a 96-well plate. After inverting the plate for 4 h, medium was added, and cells were cultured for 4 days. The medium was replaced with 100 *μ*L of serum-free medium (DMEM/F-12 supplemented with 1% penicillin/streptomycin) containing 0, 10, 20, or 30 *μ*M U0126. Then, the cells were incubated with U0126 for 24 h, the medium was discarded, cells were rinsed with PBS, and 100 *μ*L per well of medium containing 10% CCK-8 reagent was added to each well. A microplate reader was used to measure the OD value of each well at 450 nm after 2 h of reaction.

### 2.12. Elastin Content Measurement

A Fastin Elastin Assay Kit (Biocolor, UK, Cat.No.F2000) was employed according to the manufacturer's protocol for elastin analysis. Briefly, PGA-VSMCs were digested with 0.25 M oxalic acid at 100°C 2 h and elastin was extracted with a precipitating reagent, collected by centrifugation at 10 000 ×*g*, and the pellet was mixed with 250 *μ*l Fastin dye reagent for 90 min. The elastin dye was then mixed with a 250 *μ*l dissociation reagent for 15 min under gentle agitation in the dark. The absorbance of recovered dye, standards, and blank was recorded at 512 nm, using a microplate reader.

### 2.13. Statistical Analysis

GraphPad Prism (version 8.0) was used to conduct all statistical analyses. The analysis was performed for three independent experiments and three technical replicates for each sample. The mean and standard deviation were used to represent the data. Differences between two groups were compared by two-tailed, unpaired Student's *t*-test. For multiple group comparisons, one-way analysis of variance (one-way ANOVA) followed by Turkey's post-test was used or two-way analysis of variance followed by Bonferroni's post-test was used. Statistical significance was defined as a *p* value of less than 0.05.

## 3. Results

### 3.1. Human Vascular Smooth Muscle Cells on a PGA Scaffold Culture System

**Figure 1:**
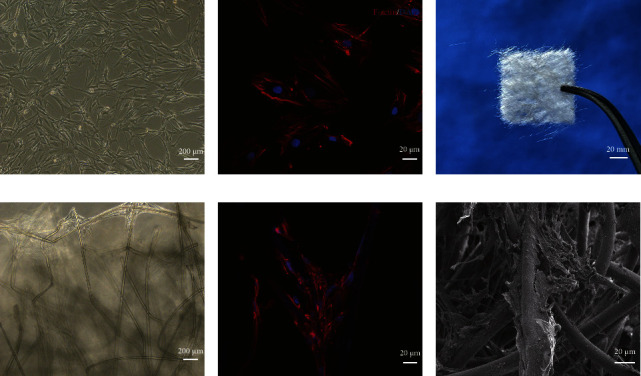
Morphology of seed cells and PGA-VSMCs. (a) Microscope image and (b) fluorescent microscope images of seed cells. (c) General view of the PGA nonwoven fabric. (d) Microscope image, (e) fluorescent microscope image, and (f) SEM images of PGA-VSMCs. The cell nucleus is represented by blue dots, and F-actin is represented by red areas.

**Figure 2:**
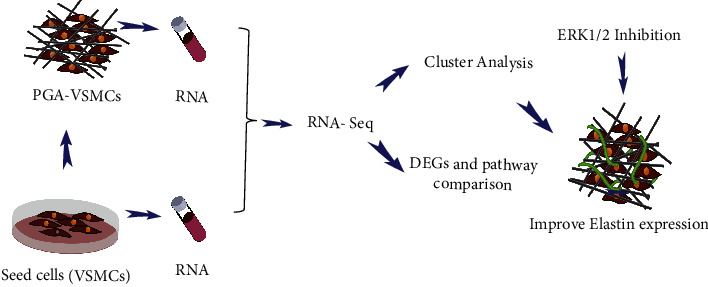
Schematic representation of the experimental design.

The PGA nonwoven fabric scaffold material was the same as that used in our previous TEBV study (Figure 1(c)) [5, 6]. The relevant parameters of the PGA materials are shown in Figure S1. The fiber diameter of the PGA was about 15 *μ*m, the porosity was 63.18%, the relative molecular weight was 47 kDa, and the molecular weight distribution range was 24–60 kDa. The PGA scaffold material had almost no degradation in the first 11 days and maintained certain mechanical properties. The high porosity of a PGA provided sufficient surface area to support cell adhesion and growth. Human VSMCs were derived from the aorta of healthy donors using the patch method. The cell identification of VSMCs is shown in Figure S2. The VSMCs used in seed cells and PGA-VSMCs were all from the same healthy donor. Cells from a different donor were used for each independent experiment. The experimental design scheme is briefly shown in Figure 2. By observing the skeleton structure (F-actin) of the cells, it was found that the seed cells grew flat and spread on a plane (Figures 1(a) and 1(b)). In the PGA-VSMC culture, cells grew along PGA fibers. Cells also grew by establishing connections in the spatial structure between fibers, showing a 3D state (Figures 1(d)–1(f)).

### 3.2. Transcriptome Analysis of Seed Cells and PGA-VSMCs

**Figure 3:**
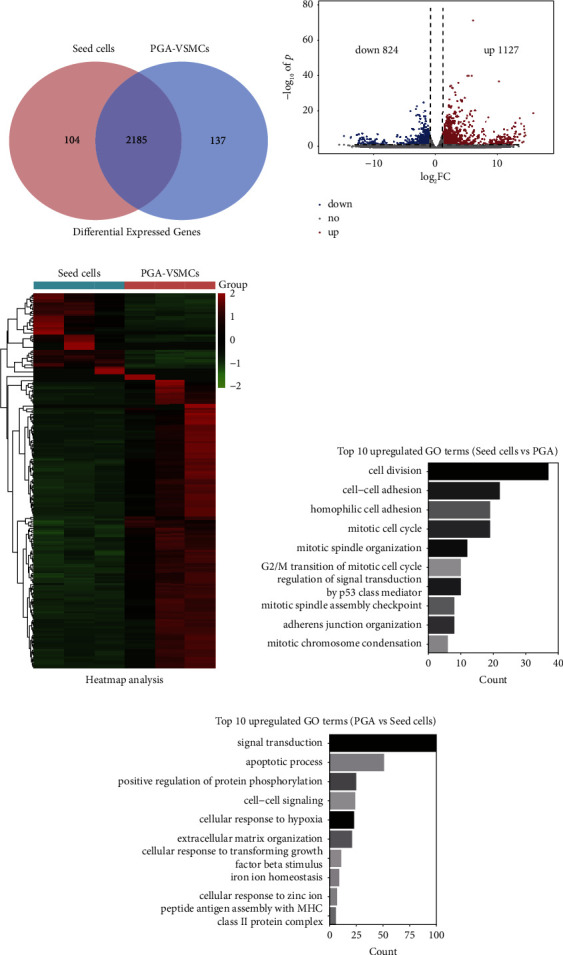
Transcriptome data analysis. (a) The genes identified in seed cells and PGA-VSMCs are shown in a Venn diagram. (b) Volcano plot of the 1961 DEGs. Red DEGs with fold change ≥1.2; blue DEGs with fold change ≤−1.2. (c) Heatmap of the 1961 DEGs in seed cells and PGA-VSMCs. (d) and (e) Top 10 GO terms of upregulated genes in seed cells and PGA-VSMCs.

Next, we used transcriptome sequencing to study the gene expression of seed cells and PGA-VSMCs. We identified 2,289 and 2,322 genes in seed cells and PGA-VSMCs, respectively. There were 2,185 genes that were expressed in both groups, with 104 genes only expressed in seed cells and 137 genes only expressed in PGA-VSMCs (Figure 3(a)). The fold change in the identified genes was calculated by combining the filtered reads with the FPKM value. For genes expressed in both the groups, |log_2_ FC| ≥1.2 and *p* value <0.05 were used as cut-off values for identifying DEGs, using the DEseq2 R package. A total of 1961 DEGs in the seed cells and PGA-VSMCs combined were identified. Among them, 1,131 genes were upregulated and 830 genes were downregulated (Figures 3(b) and 3(c)). Among these DEGs, we detected the RNA expression of 10 genes through quantitative reverse transcriptase-polymerase chain reaction (RT-qPCR), and their expression levels matched the transcriptome analysis results. RT-qPCR verified the validity of the sequencing data, as shown in Figure S3. In addition, we performed a functional analysis of the DEGs in seed cells and PGA-VSMCs. Genes upregulated in seed cells were mainly enriched in the cell division and cell-cell adhesion (Figure 3(d)). The genes upregulated in PGA-VSMCs were mainly enriched in signal transduction, apoptotic process, and positive regulation of protein phosphorylation (Figure 3(e)). These results suggest that the seed cell culture promoted cell proliferation and cell-cell adhesion. The 3D PGA culture promoted cell adaptation to the growth environment through signal transduction. Using the quantitative RT-PCR analysis, we found that seed cells had higher expression of the contractile VSMC genes such as *α*-SMA, Calponin, SMMHC, and PGA-VSMCs had higher expression of the synthetic VSMC genes such as OPN. These results indicate that seed cells are closer to a contractile phenotype, and PGA-VSMCs are closer to a synthetic phenotype (Figure S4).

### 3.3. Network and Cluster Analyses of the DEG Protein-Protein Interaction Network (PPI)

**Figure 4:**
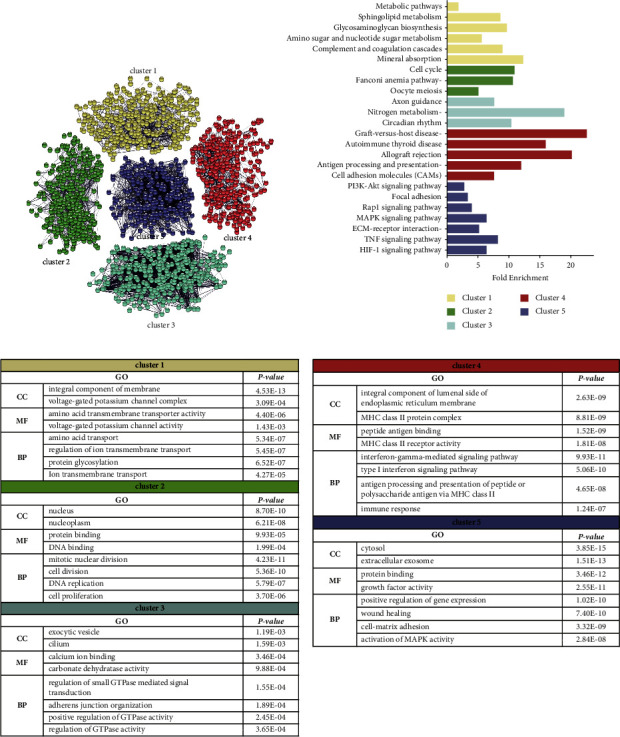
Network and cluster analysis of the DEG protein-protein interaction network (PPI). (a) Network and cluster analysis of DEGs. (b) Kyoto encyclopedia of genes and genomes (KEGG) pathway terms for five clusters. (c) GO annotation analysis for five clusters.

To investigate the function of the DEGs, we performed a network and cluster analysis on the DEGs using the STRING database, which is a database of protein-protein interactions curated from major experimental findings repositories. A total of five clusters were obtained through the *k*-means method of the STRING database, and these five clusters were named clusters 1, 2, 3, 4, and 5 (Figure 4(a)). KEGG (Figure 4(b)) and GO (Figure 4(c)) analyses were performed on each cluster using the DAVID database. We used the Cytoscape tool cytoHubba to calculate the rank of hub genes and showed the expression of hub genes in the form of heatmaps (Figure S5).

In cluster 1, the GO component analysis showed that the DEGs were mainly membrane proteins and ion channel complexes. Solute carrier family genes were downregulated, and KCND2 expression was upregulated. In seed cells and PGA-VSMCs, there were differences in cellular material transport and metabolism. In cluster 2, DEGs were enriched in the nucleus and were mainly involved in cell cycle-related regulation, protein binding, and DNA binding. All hub genes in cluster 2 were downregulated. In PGA-VSMCs, cell proliferation slowed. In cluster 3, DEGs were enriched in the exocytic vesicles and cilium. *DNAH11*, *CCDC151*, *TTC25*, and *RSPH9* related to ciliary structure composition were upregulated, and genes affecting cilia motility were downregulated. Changes in cilia-related genes were found in PGA-VSMCs. In cluster 4, DEGs were mainly immune-related molecules, such as major histocompatibility complex (MHC) protein complexes, and were involved in the secretion of vesicles, antigen processing, and the positive regulation of immune system processes. MHC genes were upregulated, and *STAT1* was downregulated. In cluster 5, DEGs were mainly involved in the cytosol, extracellular exosome, and extracellular space, which are closely related to the tissue development. In addition, there were many pathways enriched by this cluster, including MAPK, PI3K-AKT, and other signaling pathways related to growth and development. The top ten hub genes were cytokines (*IL6*, *VEGFA*, *EGF*, *HGF*, *CTGF*, and *TGFB2*), matrix metalloproteinases (*MMP9* and *TIMP1*), *CD44*, and actin alpha 2 (*ACTA2*). In PGA-VSMCs, vascular growth regulators and remodeling factors were activated.

### 3.4. Elastin and ERK1/2 Signaling Pathways of Seed Cells and PGA-VSMCs

**Figure 5:**
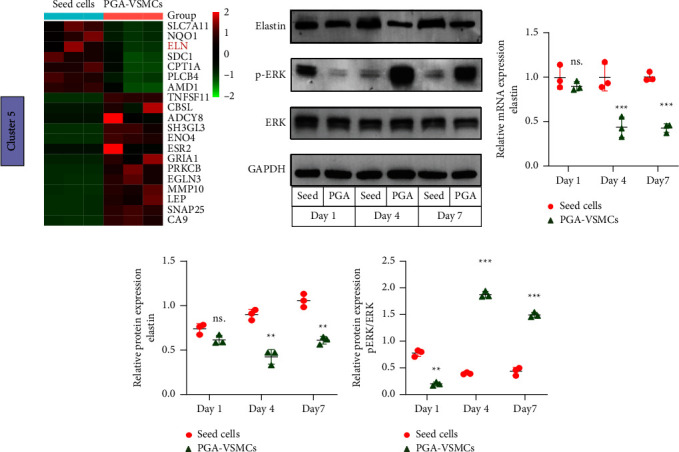
Elastin and ERK1/2 signaling pathway analysis of seed cells and PGA-VSMCs. (a) Heatmap analysis of the top genes in cluster 5. (b) Western blot gels of phosphorylated-ERK1/2 (p-ERK), total ERK1/2 (t-ERK) and elastin. (c) RT-qPCR analysis of elastin mRNA levels in seed cells and PGA-VSMCs (*p*=0.5946, *p* < 0.001, *p* < 0.001). (d) Protein levels of elastin in seed cells and PGA-VSMCs (*p*=0.1440, *p*=0.0055, *p*=0.0065). (e) Protein levels of p-ERK/t-ERK in seed cells and PGA-VSMCs (*p*=0.0018, *p*=0.0006, *p*=0.0003). Mean ± SD, *n* = 3 (^*∗∗*^*p* < 0.01, ^*∗∗∗*^*p* < 0.001).

The mechanical characteristics of TEBVs are dependent on the secretion of ECM. Dahl et al. found that TEBVs were rich in collagen, but lacked elastin [7]. Moreover, we found that collagen secretion was not reduced (RT-qPCR data are in Figure S6, and sequencing data are in Table S2), but the expression of elastin was reduced (Figure 5(a)). Elastin-related pathways were found by a GeneCards search, including the integrin pathway, ERK signaling pathway, and phospholipase C Pathway (Table 1). Some studies indicated that the ERK1/2-MAPK signaling pathway was closely related to the synthesis of elastin [20]. In our study, the MAPK signaling pathway was activated in cluster 5 (Figure 4(b)). Based on these findings, we then explored the relationship between the ERK1/2 signaling pathway and elastin synthesis in PGA-VSMCs. Significant decreases in elastin mRNA and protein levels were detected in the PGA-VSMCs on days 4 and 7 compared with the seed cells (Figures 5(a)–5(d)). Western blotting was used to measure the amount of phosphorylation of ERK1/2 (Figures 5(b) and 5(e)), and it was found that the expression in PGA-VSMCs increased significantly on days 4 and 7. These results indicated that activation of ERK1/2-MAPK signaling may be an upstream regulator of elastin secretion.

### 3.5. Inhibition of ERK1/2 Phosphorylation Increases Elastin Synthesis in PGA-VSMCs

**Figure 6:**
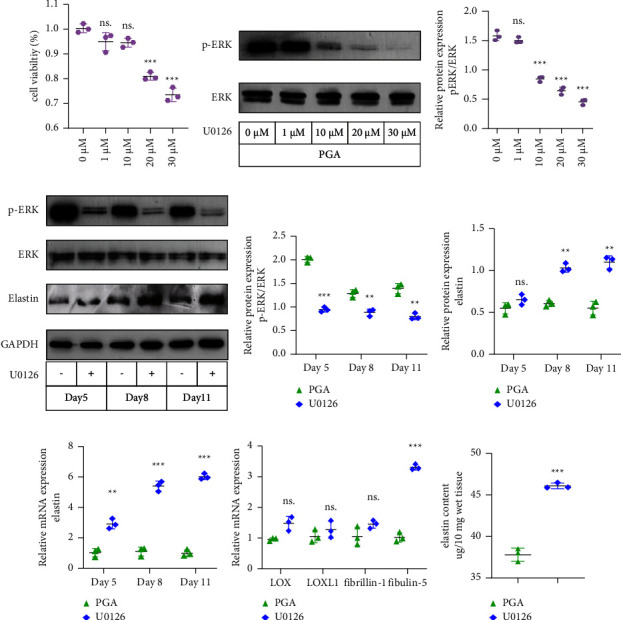
Inhibition of ERK1/2 phosphorylation increases elastin synthesis in PGA-VSMCs. (a) The effects of U0126 gradient concentrations on the viability of VSMCs as determined by a CCK-8 assay (*p*=0.01199, *p*=0.0676, *p* < 0.001, *p* < 0.001). (b, c) Protein levels of p-ERK in VSMCs treated with 0, 1, 10, 20, and 30 *μ*Μ U0126 (*p*=0.3566, *p* < 0.001, *p* < 0.001, *p* < 0.001). (d) Western blot gels of pERK, tERK and elastin in PGA-VSMCs with or without U0126 treatment for 5, 8, and 11 days. (e) Protein levels of pERK/tERK in VSMCs treated for 5, 8 and 11 days (*p* < 0.001, *p*=0.0095, *p*=0.0075). (f) Protein levels of elastin in VSMCs treated for 5, 8 and 11 days (*p*=0.3117, *p*=0.0016, *p*=0.0032). (g) RT-qPCR analysis of elastin in PGA-VSMCs with or without U0126 treatment for 5, 8, and 11 days (*p*=0.0052, *p*=0.0002, *p* < 0.001). (h) RT-qPCR analysis of LOX, LOXL1, fibrillin-1 and fibulin-5 mRNA levels in PGA-VSMCs with or without U0126 treatment for 5 days (*p*=0.0613, *p*=0.1532, *p*=0.1913, *p*=0.0009). (i) Elastin contents (*μ*g/10 mg of total wet weight) of PGA-VSMCs with or without U0126 treatment for 11 days (*p* < 0.001). Mean ± SD, *n* = 3 (^*∗∗*^*p* < 0.01, ^*∗∗∗*^*p* < 0.001).

**Figure 7:**
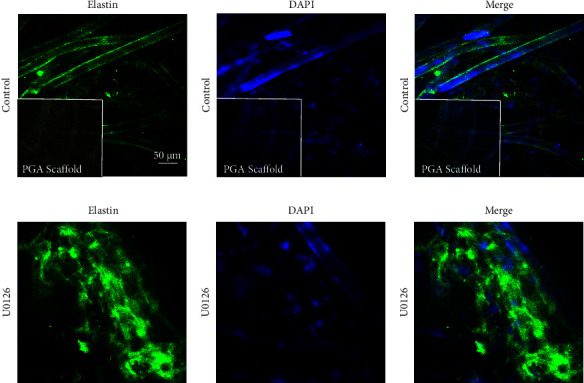
Immunofluorescence images of elastin expression after inhibition of ERK1/2 phosphorylation. (a–c) Immunofluorescence staining of PGA-VSMCs without U0126. (d–f) Immunofluorescence staining of PGA-VSMCs with U0126 added; green is elastin, nuclei are in blue; scale bar = 50 *μ*m.

To analyze whether the reduction in elastin in PGA-VSMCs required the activation of ERK1/2, we examined the effect of treating PGA-VSMCs with U0126 (an ERK1/2 phosphorylation inhibitor) on the inhibition of elastin gene transcription. Because the phosphorylation levels of ERK1/2 in PGA-VSMCs increased on the fourth day, the starting point of U0126 treatment detection was day 4 of the culture. First, we assessed the effect of U0126 on cytotoxicity. PGA-VSMCs cultured for 4 days were treated with 0, 1, 10, 20, or 30 *μ*M U0126 for 24 h, and the cell viability was determined by a CCK-8 assay (Figure 6(a)). The results showed that at U0126 concentrations between 0 and 10 *μ*M, no obvious effect on the viability of PGA-VSMCs was observed. In addition, we explored the inhibitory effect of 0, 1, 10, 20, and 30 *μ*M U0126 treatment on phosphorylation of ERK1/2 in PGA-VSMCs for 5 days (Figures 6(b) and 6(c)). At U0126 concentrations from 10 to 30 *μ*M, the inhibitory effect on phosphorylation of ERK1/2 was found to be statistically significant. Therefore, we selected the treatment of PGA-VSMCs with 10 *μ*M U0126 for subsequent cultures (Figures 6(d) and 6(e)). The results showed that PGA-VSMCs with U0126 increased the mRNA expression levels of elastin on days 5, 8, and 11 (Figure 6(g)). The protein level of elastin in PGA-VSMCs was found to be statistically significant on days 8 and 11 (Figures 6(d) and 6(f)). Elastin is an ECM protein secreted by VSMCs, which are mainly distributed around the cells. We also examined the secretion of elastin in PGA-VSMCs by the Fastin Elastin Assay Kit and immunofluorescence. We found that U0126 inhibitor promoted the secretion of elastin in PGA-VSMCs (Figure 6(i), Figure 7). In addition, we observed the expression levels of proteins encoded by genes that constitute elastic fibers (fibrillin-1 and fibulin-5) or cross-linking proteins of elastin monomers (*LOX* and *LOXL1*). We found that the expression of fibulin-5 was increased, and the expression of fibrillin-1, *LOX*, and *LOXL1* was not affected (Figure 6(h)). As a result, inhibiting ERK1/2 phosphorylation appears to be a promising technique for increasing elastin production.

## 4. Discussion

Previous studies have shown that the biological behavior of VSMCs was variable and easily affected by scaffold materials. Although TEBVs based on PGA scaffolds have achieved a certain level of success, there is still a lack of research on the biological effects of PGA materials on cells and on the lack of elastin secretion in the culture of TEBVs. We transferred seeded cells to 3D PGA scaffolds and then performed a transcriptomic comparison of seed cells versus PGA-VSMCs. The results of the hierarchical cluster analysis showed that seed cells and PGA-VSMCs had differences in cellular material transport and metabolism, cell proliferation, cilia-related genes, immune regulation, vascular growth regulators, and remodeling factors. Moreover, it was found that the ERK1/2-MAPK signaling pathway was activated in PGA-VSMCs. U0126 inhibited ERK1/2 phosphorylation, which increased the expression of elastin in PGA-VSMCs.

After the seed cells were transferred to the PGA scaffold, the most intuitive finding was that the morphology of the cells changed. It has been shown that the traction force exerted by cells through their interaction with the scaffold material affects cytoskeletal tension. Changes in cell morphology and associated signaling cascades are then induced. Ultimately, gene expression is altered to regulate cell function and tissue development and regeneration [21]. For changes in cell function, we used transcriptome sequencing as verification. The results showed that after the seed cells were transferred to the PGA scaffold, 1127 genes were upregulated and 824 genes were downregulated. The functional analysis of these differential genes yielded multilevel results. For example, cilia-related gene expression was altered. It has been suggested that the primary cilia of VSMCs have mechanical and ECM sensing properties and may represent one of the specific sensory sites in the vasculature [22]. Cells cultured in PGA scaffolds may sense material changes through cilia. Furthermore, it was found in our study that cell-cell adhesion-related genes were significantly upregulated in seed cells. VSMCs are adherent cells that need to adhere to the culture material. There are two main types of cell adhesion: cell-cell adhesion and cell-ECM adhesion. Cells can sense external forces and geometric constraints through these interactions [23]. Our results suggested that cell-cell adhesion was more predominant in seed cells, and further proof is needed in the future as to whether cell-ECM adhesion is more predominant in PGA-VSMCs. In addition, researchers have linked the slowed proliferation in 3D cultures to differences in cell adhesion. Li et al. [24] found that the slower cell proliferation in 3D scaffold cultures might be related to the decreased expression of focal adhesion kinase phosphorylation, which led to increased P21 expression and ultimately slowed cell proliferation [25]. The PGA scaffold has been reported to induce apoptosis to inhibit cell proliferation [26]. Some studies have tried to reform PGA, such as using hyaluronic acid coating to improve biocompatibility; promote cell adhesion by using peptide interfacial biomaterials [27, 28]. We will further study how to improve the scaffold materials to reduce cell apoptosis. According to the traditional definition in 2D culture, the proliferation of VSMCs with synthetic phenotype should be significantly increased, but the expression of PGA-VSMCs in our results was significantly decreased. Others have also found that in 3D collagen I gel, compared to 2D culture, cell proliferation is greatly reduced, but the expression of contractile protein *α*-SMA is downregulated [29]. They believe that the reasons are the influence of the material space and the extracellular matrix, and the definition of 3D and 2D may be different. We suspect that this phenomenon of PGA-VSMCs may be related to degradation products [30] and the spatial structure of PGA. More experiments may be needed to study the phenotype of VSMCs. For PGA-guided tissue regeneration, we found that PGA-VSMCs activated factors associated with vascular remodeling. When VSMCs remodel, cytokines are required to promote matrix regeneration [31]. In addition, metal matrix proteases are required to remodel the ECM [32]. PGA scaffolds support the growth of VSMCs and tissue regeneration and ultimately form tissue-engineered blood vessels that can be used in clinical applications [3]. However, they fail to escape the activation of human leukocyte antigen (HLA), similar to most polymer materials [33]. However, the TEBVs we transplanted into the body were cultured for 8 weeks. The PGA scaffolds were basically degraded during this period and therefore would not cause immune rejection in the body. As for the effect of HLA activation on tissue regeneration in vitro, this remains to be determined by subsequent experiments.

Our results showed that the DEGs were mainly ECM-related regulatory proteins in cluster 5. When VSMCs are cultured in traditional tissue culture dishes, they are known to secrete both soluble and insoluble elastin. However, a change in culture conditions from seed cells to 3D porous scaffolds leds to a loss of elastin biosynthesis in VSMCs in past studies [18]. Some studies have found that the decrease of elastin secretion is related to the decrease of the elastin mRNA steady-state level [19]. For the regulation of elastin synthesis, the most studied mechanism may be the ERK1/2 signaling pathway that reduces elastin gene transcription [34–37]. Researchers have also demonstrated that inhibition of ERK1/2 phosphorylation increased elastin synthesis in VSMCs *in vitro* and in the aorta *in vivo* [20]. Therefore, we attempted to inhibit ERK1/2 phosphorylation in PGA-VSMCs in vitro and also confirmed that the level of elastin increased in PGA-VSMCs. Inhibition of ERK1/2 phosphorylation levels is therefore a promising strategy for regulating elastin synthesis in TEBVs. Considering the effect of ERK inhibitors on cell proliferation [38], we intend to try to add ERK1/2 inhibitor in the later period of tissue engineering culture (noncell proliferation period) in the future to reduce the influence of cell proliferation inhibition on TEBVs.

Identifying transcriptomic changes in cells early in culture can enhance our understanding of biomaterial-cell interactions and help guide future use and design of materials. This study helped us to further understand the mechanisms of TEBVs cultured in bioreactors by analyzing the gene expression of cells in a small PGA culture model. Although the scaffold material is a key factor affecting TEBVs, factors such as mechanical stimulation and medium composition can also affect the growth of TEBVs, which should be considered in future studies. In addition, this study provided a new perspective on *in vitro* tissue engineering culture and provided valuable insights for the improvement of TEBVs.

## 5. Conclusion

In this study, we used high-throughput methods to compare PGA-VSMCs with seed cells. It was found that seed cells promoted cell proliferation and cell-cell adhesion. The 3D PGA culture helped cells adapt to the growth environment through signal transduction. Moreover, we found that the ERK1/2-MAPK pathway was continuously activated in the 3D PGA environment and was highly correlated with the secretion of elastin. In conclusion, we elucidated how PGA scaffolds affect the growth of VSMCs and successfully increased the secretion of elastin.

## Figures and Tables

**Table 1:** Pathways affecting elastin synthesis in the GeneCards network.

Pathway	Contained pathways	Similarity score
Integrin pathway	FAK1 signaling	0.67
	Integrin pathway	0.67
	GnRH signaling	0.56

ERK signaling	ERK signaling	0.61
	Rho family GTPases	0.61
	MAPK signaling	0.58

Phospholipase C pathway	Phospholipase C pathway	0.56

Extracellular matrix organization	Degradation of the extracellular matrix	0.47

Elastic fiber formation	Elastic fiber formation	0.84

## Data Availability

The data underlying this article are available in the Gene Expression Omnibus (GEO) Database at https://www.ncbi.nlm.nih.gov/geo/query/acc.cgi?acc=GSE192986 and can be accessed with GSE192986.

## Supplementary Materials

Supplementary Materials Figure S1. Characterization of PGA Scaffold. Figure S2. Identification of VSMCs. Figure S3. The validity of sequencing was tested by RT-qPCR. Figure S4. Analysis of VSMC phenotype markers. Figure S5. Heatmap of hub genes in 5 clusters. Figure S6. The mRNA expression of collagen 1 and collagen 3. Table S1. Primers of real time RT-qPCR. Table S2. Sequencing results of collagen 1 and collagen 3.
