# Supplementary material for: Transcriptome Analysis of Human Vascular Smooth Muscle Cells Cultured on a Polyglycolic Acid Mesh Scaffold
Source: J Tissue Eng Regen Med. 2023 Jun 22;2023:9956190. doi: 10.1155/2023/9956190 (PMC11919212; doi:10.1155/2023/9956190)
Supplement: Supplementary Materials — Figure S1. Characterization of PGA Scaffold. Figure S2. Identification of VSMCs. Figure S3. The validity of sequencing was tested by RT-qPCR. Figure S4. Analysis of VSMC phenotype markers. Figure S5. Heatmap of hub genes in 5 clusters. Figure S6. The mRNA expression of collagen 1 and collagen 3. Table S1. Primers of real time RT-qPCR. Table S2. Sequencing results of collagen 1 and collagen 3. [file 9956190.f1.zip › Supplementary-table S1.docx]

**Table S1 primers of real time qPCR**

| **Gene name** | **Forward (5′–3′)** | **Reverse (5′–3′)** |
| --- | --- | --- |
| **VEGFA** | TCACAGGTACAGGGATGAGGACAC | TCCTGGGCAACTCAGAAGCA |
| **FGF9** | ATGGCTCCCTTAGGTGAAGTT | TCCGCCTGAGAATCCCCTTT |
| **TIMP1** | GGGCTTCACCAAGACCTACA | TGCAGGGGATGGATAAACA |
| **COL I** | GCCAAGACGAAGACATCCCA | CACCATCATTTCCACGAGCA |
| **COL III** | AATCAGGTAGACCCGGACGA | CTCCTGGGATGCCATTTGGT |
| **ELN** | GAGCTTTTGCTGGAATCCCA | GGCAGTTTCCCTGTGGTGTAG |
| **MMP9** | GGGCTCCCGTCCTGCTT | CCTCCCTTTCCTCCAGAACA |
| **CDH6** | AAGGAGTTTTACACAGCCACTGT | CTTGGTACTGCTCCCTGTTTTCT |
| **IL6** | CTGGATTCAATGAGGAGAC | AATCTGTTCTGGAGGTACT |
| **MKI67** | AAGAAGAGGTCCTACCAGTCGG | CCTACCAGTTCCATAGTTTGC |
| **ITGA3** | TCAACCTGGATACCCGATTCC | GCTCTGTCTGCCGATGGAG |
| **ACTA2** | TCCGGGACTACAAGGAGAACT | CCCATCAGGCAACTCGTAACTC |
| **LOX** | CAGAGGAGAGTGGCTGAAGG | CCAGGACTCAATCCCTGTGT |
| **LOXL1** | TGGCTGAACTCGTCCATGCTGTG | ACTACGATGTGCGGGTGCTACTG |
| **fibrillin-1** | GCGGAAATCAGTGTATTGTCCC | CAGTGTTGTATGGATCTGGAGC |
| **fibulin-5** | TCGCTATGGTTACTGCCAGCA | TTGGCAAGACCTTCCATCGTC |
| **Calponin** | GGCCCAGAAGTATGACCACC | CCATCTTTGAGGCCGTCCAT |
| **SMMHC** | GCCTACGGAGAGCTGGAAAA | AATGTTGGCTCCCACGATGT |
| **OPN** | AGCAGAATCTCCTAGCCCCA | ACGGCTGTCCCAATCAGAAG |
| **GAPDH** | GGTGGTCTCCTCTGACTTCAACA | GTTGCTGTAGCCAAATTCGTTGT |
