# Supplementary material for: Transcriptome Analysis of Human Vascular Smooth Muscle Cells Cultured on a Polyglycolic Acid Mesh Scaffold
Source: J Tissue Eng Regen Med. 2023 Jun 22;2023:9956190. doi: 10.1155/2023/9956190 (PMC11919212; doi:10.1155/2023/9956190)
Supplement: Supplementary Materials — Figure S1. Characterization of PGA Scaffold. Figure S2. Identification of VSMCs. Figure S3. The validity of sequencing was tested by RT-qPCR. Figure S4. Analysis of VSMC phenotype markers. Figure S5. Heatmap of hub genes in 5 clusters. Figure S6. The mRNA expression of collagen 1 and collagen 3. Table S1. Primers of real time RT-qPCR. Table S2. Sequencing results of collagen 1 and collagen 3. [file 9956190.f1.zip › Supplementary-table S2.docx]

**Table S2 Sequencing results of collagen 1 and collagen 3**

| **Gene ID** | **Gene Name** | **log_2_(FC)** | **P value** | **significant** |
| --- | --- | --- | --- | --- |
| ENSG00000164692 | COL1A2 | 0.2 | 0.42 | no |
| ENSG00000108821 | COL1A1 | -0.18 | 0.67 | no |
| ENSG00000168542 | COL3A1 | 1.64 | 0 | yes |
