# Supplementary figures and images for: Transcriptome Analysis of Human Vascular Smooth Muscle Cells Cultured on a Polyglycolic Acid Mesh Scaffold
Source: J Tissue Eng Regen Med. 2023 Jun 22;2023:9956190. doi: 10.1155/2023/9956190 (PMC11919212; doi:10.1155/2023/9956190)

A

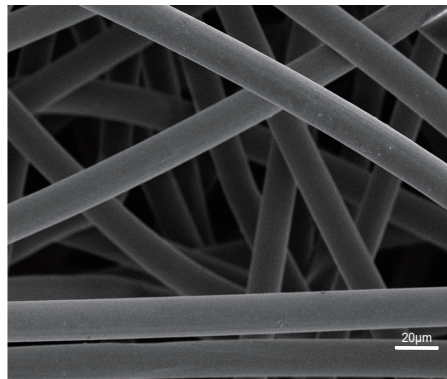

B

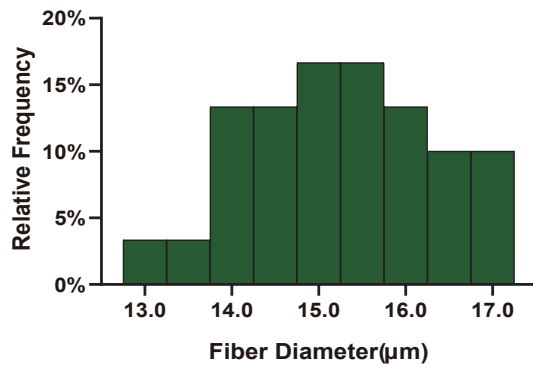

C

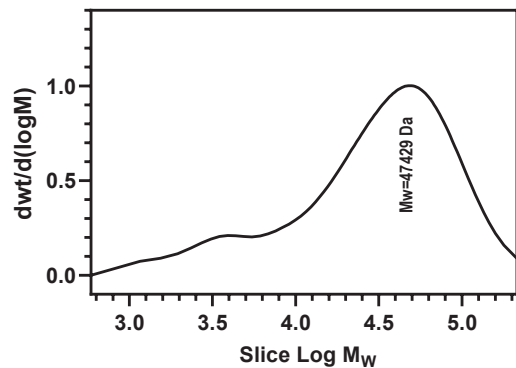

D

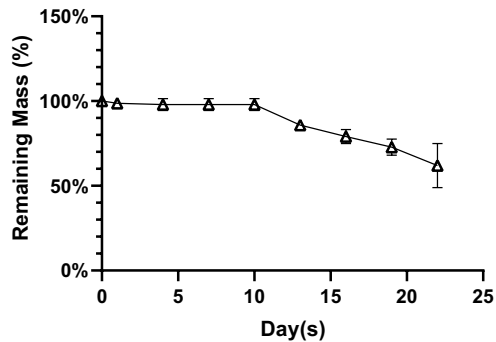

Supplement: Supplementary Materials — Figure S1. Characterization of PGA Scaffold. Figure S2. Identification of VSMCs. Figure S3. The validity of sequencing was tested by RT-qPCR. Figure S4. Analysis of VSMC phenotype markers. Figure S5. Heatmap of hub genes in 5 clusters. Figure S6. The mRNA expression of collagen 1 and collagen 3. Table S1. Primers of real time RT-qPCR. Table S2. Sequencing results of collagen 1 and collagen 3. [file 9956190.f1.zip › supplemental-figure S1 (1).pdf]

$\alpha$ -SMA

Calponin

Merge

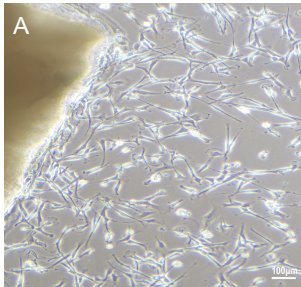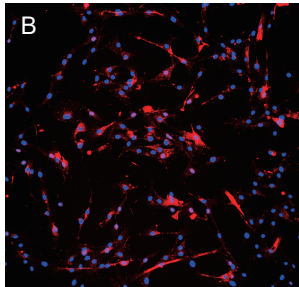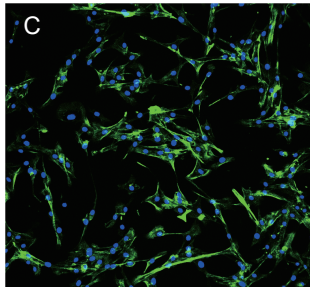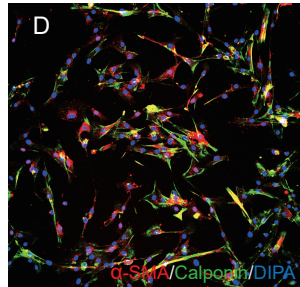

Supplement: Supplementary Materials — Figure S1. Characterization of PGA Scaffold. Figure S2. Identification of VSMCs. Figure S3. The validity of sequencing was tested by RT-qPCR. Figure S4. Analysis of VSMC phenotype markers. Figure S5. Heatmap of hub genes in 5 clusters. Figure S6. The mRNA expression of collagen 1 and collagen 3. Table S1. Primers of real time RT-qPCR. Table S2. Sequencing results of collagen 1 and collagen 3. [file 9956190.f1.zip › supplemental-figure S2 (1).pdf]

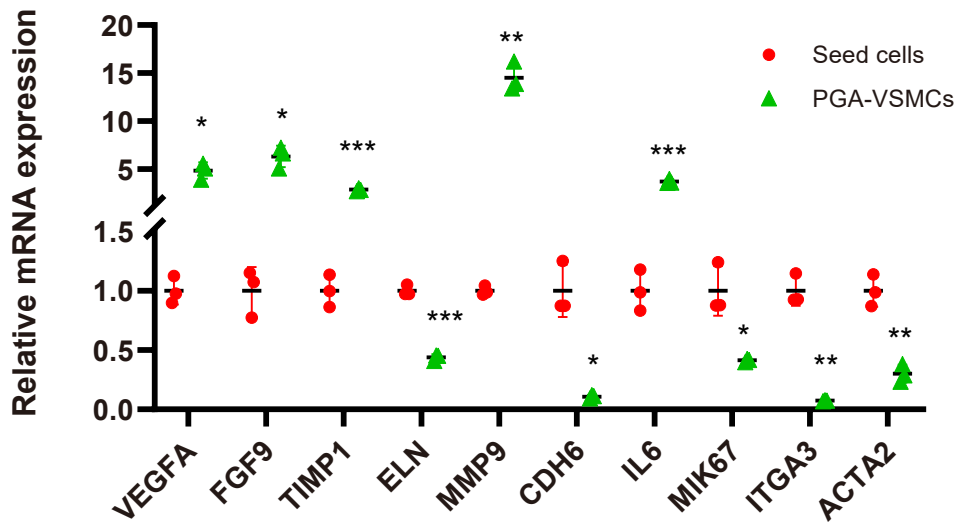

Supplement: Supplementary Materials — Figure S1. Characterization of PGA Scaffold. Figure S2. Identification of VSMCs. Figure S3. The validity of sequencing was tested by RT-qPCR. Figure S4. Analysis of VSMC phenotype markers. Figure S5. Heatmap of hub genes in 5 clusters. Figure S6. The mRNA expression of collagen 1 and collagen 3. Table S1. Primers of real time RT-qPCR. Table S2. Sequencing results of collagen 1 and collagen 3. [file 9956190.f1.zip › supplemental-figure S3 (1).pdf]

A

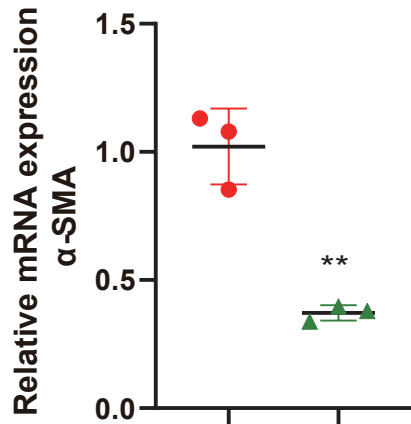

B

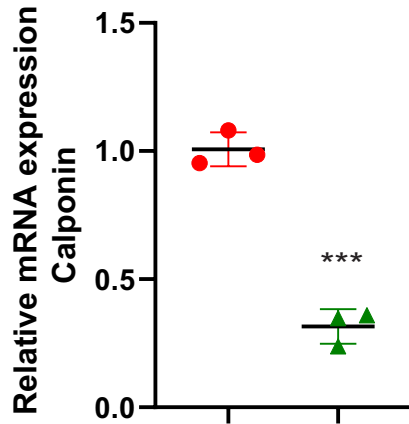

C

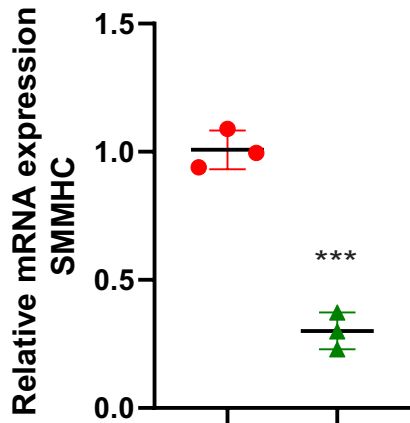

D

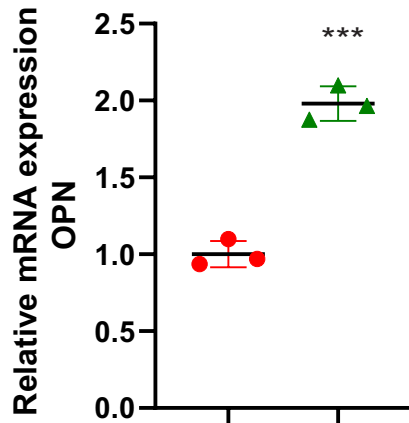

Supplement: Supplementary Materials — Figure S1. Characterization of PGA Scaffold. Figure S2. Identification of VSMCs. Figure S3. The validity of sequencing was tested by RT-qPCR. Figure S4. Analysis of VSMC phenotype markers. Figure S5. Heatmap of hub genes in 5 clusters. Figure S6. The mRNA expression of collagen 1 and collagen 3. Table S1. Primers of real time RT-qPCR. Table S2. Sequencing results of collagen 1 and collagen 3. [file 9956190.f1.zip › supplemental-figure S4 (1).pdf]

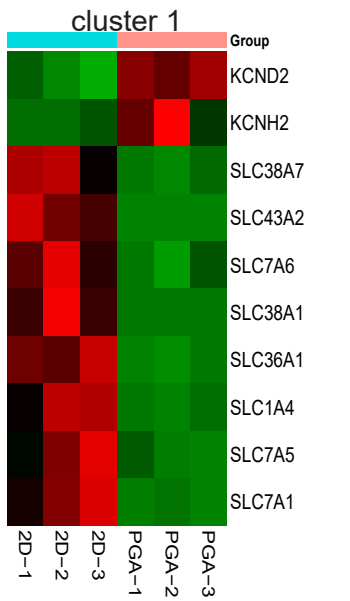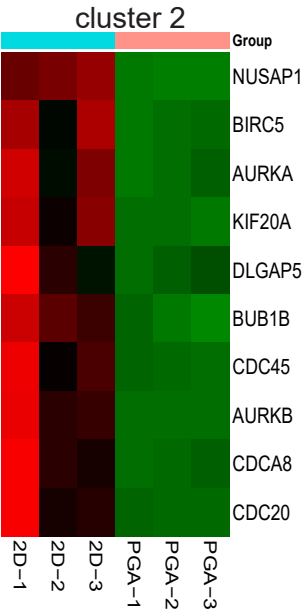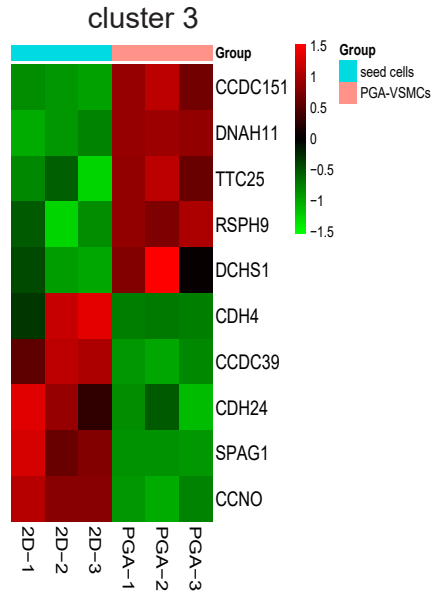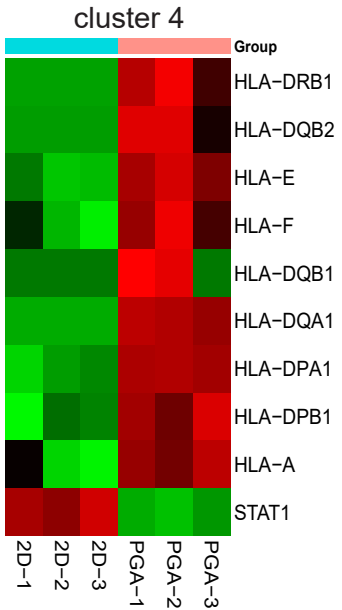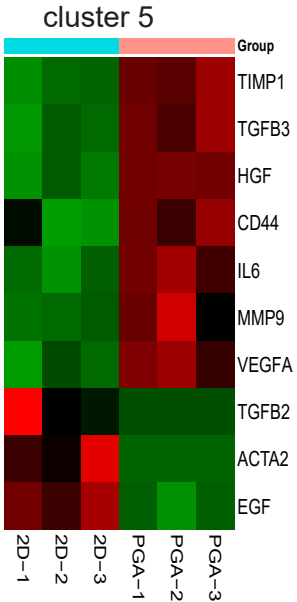

Supplement: Supplementary Materials — Figure S1. Characterization of PGA Scaffold. Figure S2. Identification of VSMCs. Figure S3. The validity of sequencing was tested by RT-qPCR. Figure S4. Analysis of VSMC phenotype markers. Figure S5. Heatmap of hub genes in 5 clusters. Figure S6. The mRNA expression of collagen 1 and collagen 3. Table S1. Primers of real time RT-qPCR. Table S2. Sequencing results of collagen 1 and collagen 3. [file 9956190.f1.zip › supplemental-figure S5 (1).pdf]

A

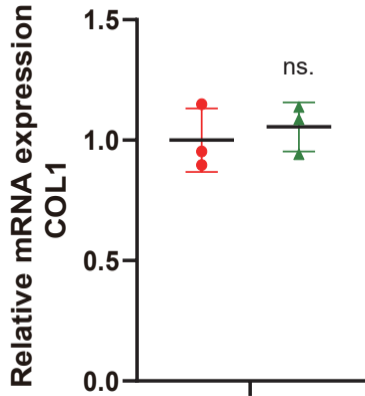

B

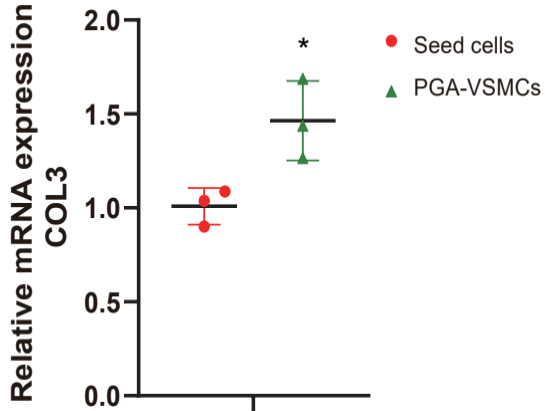

Supplement: Supplementary Materials — Figure S1. Characterization of PGA Scaffold. Figure S2. Identification of VSMCs. Figure S3. The validity of sequencing was tested by RT-qPCR. Figure S4. Analysis of VSMC phenotype markers. Figure S5. Heatmap of hub genes in 5 clusters. Figure S6. The mRNA expression of collagen 1 and collagen 3. Table S1. Primers of real time RT-qPCR. Table S2. Sequencing results of collagen 1 and collagen 3. [file 9956190.f1.zip › supplemental-figure S6.pdf]
